# Brief cognitive assessment of Alzheimer's disease in advanced stages: Proposal for a Brazilian version of the Short Battery for Severe Impairment (SIB-8)

**DOI:** 10.1590/S1980-57642013DN70200005

**Published:** 2013

**Authors:** José Roberto Wajman, Paulo Henrique Ferreira Bertolucci

**Affiliations:** 1 Behavioral Neurology Sector – Neurology and Neurosurgery Department – Federal University of São Paulo – UNIFESP, São Paulo SP, Brazil.

**Keywords:** cognitive assessment, severe impairment, dementia, Alzheimer's disease

## Abstract

The measurement of cognitive abilities of patients with severe dementia can serve a wide range of methodological and clinical needs.

**OBJECTIVE:**

To validate a proposed severe impairment battery SIB-8 for a Brazilian population sample as part of the neuropsychological assessment of patients with Alzheimer's disease (AD) in advanced stages.

**METHODS:**

After a systematic process of translation and back-translation, the SIB-8 was applied to 95 patients with AD at different stages; moderate, moderately severe and severe according to FAST subdivisions (5, 6 and 7), with scores on the Mini-Mental State Examination (MMSE) of between 5 and 15 and followed by the Division of Behavioral Neurology and the Center for Aging Brain of the Federal University of São Paulo - UNIFESP.

**RESULTS:**

Inferential data revealed that the SIB-8 instrument behaved differently at each stage of the disease with a statistical value of sensitivity p<0.001, gradually reflecting the expected course of the dementia, inherent with the decline of cognitive functions.

**CONCLUSION:**

Findings indicated that the SIB-8 is a useful tool for the evaluation and prospective comparison of AD patients in advanced stages, retaining its original characteristics in our population.

## Abstract (pt)

A quantificação da capacidade cognitiva de pacientes com demência grave pode ser útil para suprir amplas necessidades metodológicas e clínicas.

**OBJETIVO:**

Foi proposta a validação da bateria para comprometimento grave SIB-8 para uma amostra populacional Brasileira como parte integrante da avaliação neuropsicológica de pacientes com doença de Alzheimer (DA) em fases avançada.

**MÉTODOS:**

Após sistemático processo de tradução e retrotradução, a SIB-8 foi aplicada em 95 pacientes com DA nas fases moderada, moderadamente grave e grave conforme as subdivisões 5, 6 e 7 da escala FAST, com escore no Mini-Exame do Estado Mental (MEEM) entre 5 e 15 e acompanhados junto ao Setor de Neurologia do Comportamento e do Núcleo de Envelhecimento Cerebral da Universidade Federal de São Paulo - UNIFESP.

**RESULTADOS:**

Dados inferenciais revelaram que o instrumento SIB-8 comportou-se de maneira diferente em cada uma das fases da doença com valor estatístico de sensibilidade de p<0,001, relacionando gradualmente o curso esperado da demência, com o inerente declínio das funções cognitivas.

**CONCLUSÕES:**

Achados indicam a SIB-8 como uma ferramenta útil na avaliação e comparação prospectiva junto a pacientes com DA em fases avançadas, mantendo suas características originais também em nossa população.

## INTRODUCTION

The recent focus on Alzheimer's disease (AD) has created great interest in the monitoring and treatment of these patients throughout the evolutionary process of the disease. Numerous tests have been developed for the assessment of patients with dementia at preclinical, mild and moderate stages.^1^ However, toward this knowledge base, little effort has been made to quantify cognitive abilities in severely impaired patients.^2^

The measurement of these abilities in this patient group can serve a wide range of methodological and clinical needs. These data can provide an indication of preserved abilities that future health care professionals (and patients' families) can use in the administration and development of compensatory strategies. It may also allow establishment of normative data to measure current cognitive state serving as a tool for longitudinal comparison, psychological and pharmacological treatment and, finally, this knowledge can also be used in the examination of the relationship of neurochemical and neuropathological postmortem findings with cognitive status.

The development and use of standardized neuropsychological tools and properly validated diagnostic accuracy has increased and helped to characterize the cognitive decline associated with AD.^3^ However, as the disease progresses, many of the measurement instruments commonly used in neuropsychological assessment have limited applicability, which in clinical practice is explained by the so-called "floor effect" (results close to zero).

Patients are considered "untestable" when their performance on neuropsychological assessments borders the lower threshold on the scoring scale and thus patient status is considered one of generalized decline. However, these patients can retain and preserve certain skills even at more advanced stages of the disease. Authors^4^ justify the assertion that little is known about patients who are cognitively and functionally severely impaired, precisely because of the low sensitivity of the tests currently employed.

For all these reasons, more sensitive cognitive tests for more severely affected patients have been developed and are currently in use in routine clinical practice. This applies to the Test for Severe Impairment (TSI),^4^ the Modified Ordinal Scales of Psychological Development (M-OSPD),^5^ the Severe Cognitive Impairment Profile (SCIP)^6^ and the Severe Impairment Battery (SIB),^7^ among others.

Many of these tests, in fact, address the difficulties in verbal processing of these patients. However, they all require specialized training for management, as well as a range of materials for their application , and need, on average, 40 minutes for their completion. Thus, investigators^8^ compared the practical utility of five widely used neuropsychological instruments and concluded that in many criteria used as an indication of changes in cognitive state of patients with AD, tests considered "short" are good and in many instances proved better than long scales or extensive tests.

This finding helps define an ideal instrument as one that: [1] is able to clearly indicate disease progression and consequently the degree of cognitive impairment of the patient; [2] is sensitive and acceptable in terms of applicability for the language of Brazilian Portuguese; [3] is brief but assessing possible extent of higher mental functions; [4] does not require lengthy training or extensive technical and financial resources; and finally, [5] is useful for longitudinal monitoring of neuropsychological strategies and pharmacological treatments.

To this end, a short version of the Severe Impairment Battery (SIB) was devised,^9^ designed to assess cognitive impairment in severe AD patients and not amenable to clinical evaluation through the usual tests. Thus, the SIB has been specially developed to assess the adaptive and cognitive functioning of patients who are unable to complete tasks commonly used in proposed testing tools. Results of 264 patients submitted to the SIB-s, showed very high internal consistency (Cronbach's alpha = 0.97).^18^

The aim of the present study was to present a short version of the SIB applicable for use in the Brazilian population.

## METHODS

**Method for translation:** the Brazilian Portuguese version of the SIB-8 was translated applying the following methodological criteria: [1] translation by a translator with deep understanding of the instrument; [2] revision of the translation by a bilingual group involved in the research area in question; [3] review by a group representatives of the institution in which the instrument were to be applied; [4] independent back translation; and [5] evaluation of the back-translation by the bilingual group where any significant differences in their syntactic and semantic constructs were reviewed interactively. The final Brazilian Portuguese version can be found in the Appendix.

**Method of application of the battery:** the SIB is organized into nine subscales: social interaction, orientation, visuospatial ability, constructive ability, language, memory, attention, orientation to own name and praxis. Results on the original battery range from zero (0) to one hundred (100) where higher scores reveal less impairment. In this study, the 8-item version was used, whose scores range from zero to 28 points.

Although the material is presented verbally, nonverbal responses are used for the final score (2 points = correct answer, 1 point = partially correct answer and 0 = incorrect answer). The application of the battery takes approximately 15 minutes and prompting is allowed with consequent evaluation.

For this investigation, 95 subjects with moderate, moderate to severe, or severe AD were included. All subjects were followed at the Behavior Neurology Outpatients Clinic of the Federal University of Sao Paulo. The subjects' score on the Mini-Mental State Examination (MMSE^16^) had to lie between 5 and 15, Clinical Dementia Rating (CDR^17^) 2 and 3, and the Functional Assessment Staging Test (FAST), between 5 and 7. The FAST scale focuses more on an individual's level of functioning and activities of daily living (from 1: normal adult, to 7: severe dementia) versus cognitive decline.

Whenever fatigue, anxiety or nervousness were noticed the test was interrupted, the subject reassured, and the testing resumed only after the subject had calmed down, allowing for the possibility of postponing the end of the assessment until another visit.

Statistical analysis was carried out by an initial descriptive statistic with mean, median and standard deviation for quantitative variables that were plotted in dispersion graphs and a box plot. Qualitative variables were analyzed based on absolute and relative frequency calculations.

Inferential analyses were performed to confirm or refute evidence raised by the descriptive analysis: point estimation^13^ and interval^14^ of Pearson's linear correlation to quantify linear correlation. For all conclusions obtained by inferential analysis, the significance level was set as an alpha of 5%. Data were keyed into Excel 2010 for Windows for information storage and statistical analyses were performed using the software Statistical Package for the Social Sciences (SPSS) version 19.0 for Windows.

This investigation was approved by the Federal University of Sao Paulo Research Ethics Committee. An Informed Consent term was read to all subjects and their caregivers, and doubts were discussed at any time during the study.

## RESULTS

The sample in this study consisted of 95 subjects, 33 (34.7%) males and 62 (65.3%) females. The mean age was 74.7 years, ranging from 60 to 89 years, with a standard deviation of 6.2 years. The average years of schooling was 4.2 years, ranging from 3 to 8 years, with a standard deviation of 1.5 years. The mean disease duration of subjects was 7.3 years, ranging from 5 to 12 years, with a standard deviation of 1.7 years. The descriptions of all individuals according to the scales applied in this study are summarized in Table 1.

**Table 1:** Sample distribution (N and %) on CDR, FAST, MMSE and SIB-8 scales.

CDR	Moderate (2)	22	23.2%
	Severe (3)	73	76.8%
FAST	Moderate (5)	16	16.8%
	Moderately severe (6)	52	54.7%
	Severe (7)	27	28.4%
FAST	5	16	16.8%
	6A	21	22.1%
	6B	15	15.8%
	6C	16	16.8%
	7A	15	15.8%
	7B	12	12.6%
MMSE	Mean	9.6	
	Median	10.0	
	Minimum-maximum	5.0-15.0	
	Standard deviation	3.0	
SIB-8	Mean	13.8	
	Median	14.0	
	Minimum-maximum	4.0-24.0	
	Standard deviation	5.3	

**Figure 1:**
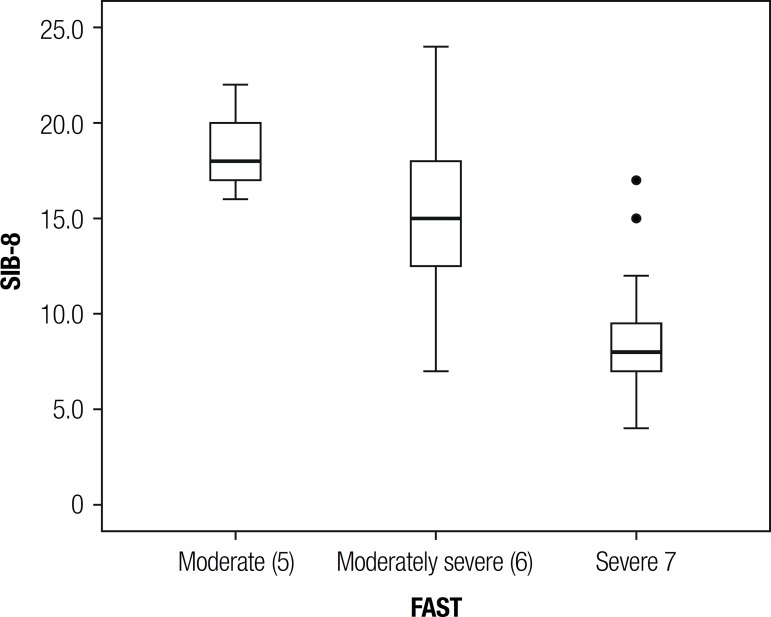
SIB-8 Boxplot according to FAST.

The FAST categories (moderate, moderately severe, severe) were evaluated with respect to scale SIB-8. Table 2 and Figure 1 gives an overview of the behavior of the SIB-8, according to the categories of FAST.

**Table 2:** SIB-8 summary measures according to FAST scale.

FAST		SIB-8
Moderate (5)	N	16
	Mean	18.4
	Median	18.0
	Minimum-maximum	16.0-22.0
	Standard deviation	2.2
Moderately severe (6)	N	52
	Mean	15.2
	Median	15.0
	Minimum-maximum	7.0-24.0
	Standard deviation	4.5
Severe (7)	N	27
	Mean	8.4
	Median	8.0
	Minimum-maximum	4.0-17.0
	Standard deviation	3.3
		
	p	<0.001

**Figure 2:**
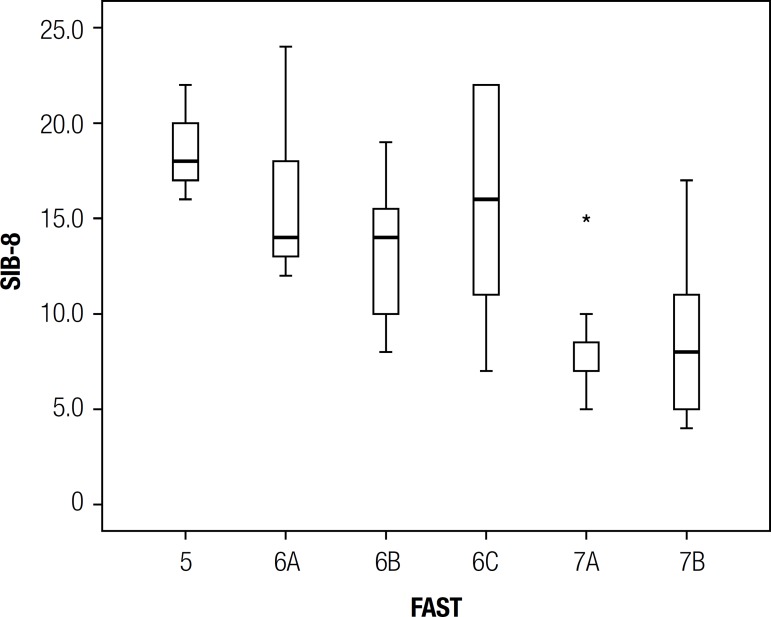
SIB-8 Boxplot according to FAST subcategories.

The FAST subcategories (5, 6A, 6B, 6C, 7A, 7B) were also evaluated for the SIB-8 scale. Table 3 and Figure 2 provide the summary measures of the behavior of the SIB-8, according to the subcategories of FAST.

**Table 3:** Summary measures of SIB-8, according to stratified FAST.

FAST		SIB-8
5	N	16
	Mean	18.4
	Median	18.0
	Minimum-maximum	16.0-22.0
	Standard deviation	2.2
6A	N	21
	Mean	16.0
	Median	14.0
	Minimum-maximum	12.0-24.0
	Standard deviation	3.5
6B	N	15
	Mean	13.1
	Median	14.0
	Minimum-maximum	8.0-19.0
	Standard deviation	3.8
6C	N	16
	Mean	16.2
	Median	16.0
	Minimum-maximum	7.0-22.0
	Standard deviation	5.7
7A	N	15
	Mean	8.5
	Median	7.0
	Minimum-maximum	5.0-15.0
	Standard deviation	2.9
7B	N	12
	Mean	8.4
	Median	8.0
	Minimum-maximum	4.0-17.0
	Standard deviation	3.9
		
	P	<0.001

The inferential results demonstrated that individuals from six different subcategories of the FAST scale did not exhibit the same scores on the SIB-8 scale (p<0.001). As highlighted and presented in summarized form in the final Table and Figure, the results of the comparisons and correlations between the sub categories revealed the findings of greater statistical value (p<0.001) according to the FAST scale functional test-moderately severe (6A) and severe (7A) stages on the FAST behaved similarly and were staggered relative to correlations with those who presented with statistically significant values.

In conclusion, in order to be clinically useful, a proposed scale must be brief and easily administered in a typical clinical practice setting. Shortened forms of the Severe Impairment Battery (SIB) have been constructed for performing evaluation and diagnosis of dementia in patients with severe cognitive impairment.^10,11^

The SIB-8 is a tool to rapidly and objectively assess cognitive change in moderate to severe AD. Clinicians may use this battery to assess the outcome of treatment and to make treatment decisions with the patient's family. To maximize the value of this tool, clinicians could administer the SIB-8 evaluation at baseline and again during subsequent clinic visits. For each assessment after baseline evaluation, a change in score can be calculated and compared with the expected rate of decline in untreated patients or according to the different types of treatment.

Successful treatment may be characterized by a modest degree of cognitive improvement or stability, but eventual decline is inevitable because of the continuous progression of the underlying disease. Stabilization at baseline levels and slower decline compared to non-treatment represent successful and desirable outcomes of treatment.^12,13^

The SIB-8 scale can be seen as a potentially useful and rapid assessment tool that may be used in clinical practice to assess patients at advanced stages of AD and their changes in cognition with disease progression or to gauge treatment response. However, this scale or any other psychometric test, cover only part of the clinical picture and cannot substitute thorough evaluation, caregiver impressions, and clinical judgment. Finally, it should be noted that the SIB alone cannot determine when to stop treatment and, to date, there are no clinical trials to guide this important decision. Nevertheless, the SIB can show clinicians that patients still have a range of abilities and that the term severe does not necessarily mean end-stage AD.^14^

Additionally, the SIB-s scale has been used in different cultures. For example, both validity and clinical utility of the SIB-s were studied for a Korean population. The test-retest correlation for the total SIB score and subscale scores were significant, except for the praxis and orienting to name. The total SIB score and subscale scores were examined according to CDR. The results suggested that the SIB can differentiate the poor performances of severely impaired dementia patients. On the basis of the receiver operating characteristic (ROC), it can be concluded that the SIB is able to accurately discriminate between CDR 2 and 3 patients. The results of this study suggest that the SIB is a reliable and valid instrument for evaluating severe dementia patients in the Korean population.^19^

The strength of this battery is the fact that it is possible to follow severely impaired patients. The instrument can also further understanding of models of disease progression, not only for purposes of didactic and academic description, but also in an attempt to find new treatments, potentially effective strategies, and cognitive or functional manipulation for intervention, aimed at improving the quality of life of these patients and their families.^15^
